# Comment on: Population preference for treatment of uncomplicated appendicitis

**DOI:** 10.1093/bjsopen/zrab114

**Published:** 2022-01-06

**Authors:** Rishi Rajesh Patel, Jared Bhaskar, Lavanya Chandel, Dhruv Gupta

**Affiliations:** St George’s University of London, London, UK; St George’s University of London, London, UK; Medical University Sofia, Sofia, Bulgaria; St George’s University of London, London, UK

*Dear Editor*

A recent population preference for treatment of uncomplicated appendicitis reported half would prefer antibiotics to surgery and that most would accept 31 per cent risk of recurrence^1^. They recommend that patients with uncomplicated appendicitis should be counselled about both antibiotic treatment alone and surgery as possible management options. The average age was 49 years in this survey but the average age of those diagnosed with appendicitis is 31 years old. It is possible that younger patients would express different choices. Appendicitis may mask underlying malignancies in older age groups so antibiotic treatment alone may be less wise in that scenario too. In the survey, 11 per cent of participants reported that they have a history of appendicitis^1^. Although this is a small proportion, their previous experience is likely to have introduced further bias.

The authors reported that 20 per cent of patients will require open surgery^1^. This statistic is outdated, with recent literature reporting just 4 per cent of patients needing an open operation^2^. It could be that participants were not accepting the risk of open surgery—nearly 30 per cent of participants preferring antibiotic treatment ranked ‘avoiding a scar’ in the top three reasons. Notably, newer trial evidence suggests lower costs and shorter off-work time for antibiotics yet surgical patients show marginal improvements in perceived quality of life (about 3–7 per cent)^3^. It is clearly important to respect patient choices irrespective of the physician views.

## References

[1] Bom WJ , Scheijmans JCG, Gans SL, Van Geloven AAW, Boermeester MA. Population preference for treatment of uncomplicated appendicitis. BJS Open 2021;5:zrab058. 34355241 10.1093/bjsopen/zrab058 PMC8411439

[2] RIFT Study Group on behalf of the West Midlands Research Collaborative. Evaluation of appendicitis risk prediction models in adults with suspected appendicitis. Br J Surg 2020;107:73–86. 31797357 10.1002/bjs.11440 PMC6972511

[3] O'Leary P, , Walsh SM, , Bolger J, , Baban C, , Humphreys H, , O'Grady S et al A Randomized Clinical Trial Evaluating the Efficacy and Quality of Life of Antibiotic-only Treatment of Acute Uncomplicated Appendicitis: Results of the COMMA Trial. *Ann Surg.* 2021;274:240–247. 33534226 10.1097/SLA.0000000000004785

